# Diabetes Mellitus, Arterial Wall, and Cardiovascular Risk Assessment

**DOI:** 10.3390/ijerph13020201

**Published:** 2016-02-06

**Authors:** Michaela Kozakova, Carlo Palombo

**Affiliations:** 1 Department of Clinical and Experimental Medicine, University of Pisa, Pisa 56122, Italy; 2 Department of Surgical, Medical, Molecular Pathology and Critical Care Medicine, University of Pisa, Pisa 56122, Italy; carlo.palombo@unipi.it

**Keywords:** diabetes, atherosclerosis, arterial stiffness, cardiovascular risk, hyperglycemia, insulin resistance

## Abstract

Diabetes mellitus is an independent risk factor for atherothrombotic cardiovascular disease. Adults with diabetes are two to four times more likely to develop heart disease or stroke than adults without diabetes. The two major features of diabetes, *i.e.*, hyperglycemia and insulin-resistance, trigger arterial stiffening and increase the susceptibility of the arterial wall to atherosclerosis at any given age. These pathological changes in the arterial wall may provide a functional and structural background for cardiovascular events. The present paper provides a critical overview of the clinical evidence linking diabetes-related metabolic abnormalities to cardiovascular risk, debates the pathophysiologic mechanisms through which insulin resistance and hyperglycemia may affect the arterial wall, and discusses the associations between vascular biomarkers, metabolic abnormalities and cardiovascular events.

## 1. Introduction

Diabetes mellitus is an independent risk factor for atherosclerosis-related cardiovascular (CV) diseases (D) [1]. In the Framingham cohort, the incidence of CVD among diabetic men and women was twice and three times that among non-diabetic men and women, respectively [2], and in a large population-based retrospective study, people with diabetes entered the high CVD risk category (a 10-year risk of 20% or more) 15 years before people without diabetes [3]. In a 25-year follow-up of middle-aged men and women, the mortality in men with diabetes and without previous coronary heart disease was equal to that of men with coronary heart disease and without diabetes (54.0 *vs.* 50.5 deaths per 1000 person-years), whereas in women with diabetes only, the risk of death was considerably higher than in women with coronary heart disease only (46.7 *vs.* 29.2 deaths per 1000 person-years) [4].

The abnormal metabolic state associated with diabetes promotes a number of alterations in the arterial tree, and subsequent vascular impairment may represent a pathophysiologic link between diabetes and CV risk. The two key metabolic abnormalities that characterize type 2 diabetes (T2DM) are hyperglycemia and insulin-resistance, and the two main pathological processes in vascular wall that can elicit CV events are atherosclerosis and arterial stiffening. From a pathologic point of view arterial stiffening, reflecting the degenerative changes of extracellular matrix (ECM) in the media layer, is distinct from atherosclerosis, a process involving the intima layer and characterized by lipid accumulation, inflammatory cells infiltration, vascular smooth muscle cells (SMCs) migration and foam cell development. Yet, the two processes often coexist in the same vascular territories, share some common risk factors and pathophysiological mechanisms and may potentiate each other in the development of vascular changes underlying CVD. Indeed, patients with diabetes mellitus show both premature atherosclerotic changes [5] and accelerated arterial stiffening [6].

The present review summarizes the clinical evidence demonstrating the link betweendiabetes-related metabolic abnormalities and CV events, debates the putative mechanisms by which hyperglycemia and insulin resistance may induce atherosclerosis and arterial wall stiffening, and discusses the role of vascular biomarkers in CV risk assessment, as well as the associations of different vascular measures with diabetes, hyperglycemia and insulin resistance.

## 2. Hyperglycemia

Hyperglycemia is the major risk factor for microvascular complications like diabetic nephropathy, retinopathy, and neuropathy [7], but its role in atherosclerosis and macrovascular disease is still under discussion [8,9]. A meta-analysis of 26 prospective studies has demonstrated that every 1% increase in HbA1c level among patients with T2DM is associated with a 17%, 15%, 11% and 29% increase in respective hazard of CV disease, coronary heart disease, stroke and peripheral arterial disease [10]. A recent study in 16,492 T2DM patients with a history of established CV disease or multiple risk factors has shown that HbA1c ≥7% is associated with a 35% increase in risk of macrovascular events [11]. Even though an association between chronic glycemic control and CV risk has been demonstrated, the studies evaluating the impact of strict glycemic control on CV events yielded controversial results. In the United Kingdom Prospective Diabetes Study (UKPDS), the intensive blood-glucose control in newly diagnosed T2DM patients, by either sulphonylureas or insulin, resulted in a long-term (10 years after the cessation of randomized interventions) risk reduction for myocardial infarction (15%), when compared with the conventional treatment group (diet) [12]. Yet, the Action to Control Cardiovascular Risk in Diabetes (ACCORD) study and Action in Diabetes and Vascular Disease: Preterax and Diamicron Modified Release Controlled Evaluation (ADVANCE) study [13] have failed to confirm this beneficial effect, probably due to the fact that T2DM patients of these trials were older and had longer duration of diabetes when the intensive glycemic control was initiated. In the STENO-2 Study, multifactorial intensive interventions targeting not only hyperglycemia, but also hypertension, dyslipidemia and microalbuminuria, reduced the risk of CV events in T2DM patients by 50% [14]. Altogether, the published data suggest that intensive treatment of hyperglycemia may result in CV benefit when initiated early in patients with short duration of diabetes, and when accompanied by treatment of other diabetes-related abnormalities, like hypertension, dyslipidemia and obesity. There is clear evidence that statins and the targeted lowering of blood pressure are each associated with substantial reduction of CV risk in patients with diabetes. A meta-analysis of 12 prospective randomized trials has demonstrated that a lipid-lowering treatment of diabetic patients reduces major coronary events by 21%, both in primary and secondary prevention [15]. In the UKPDS 38 study, a tight control of blood pressure in T2DM was associated with a clinically important reduction in the risk of myocardial infarction (21%), stroke (44%) and peripheral vascular disease (49%) [16], and in the Hypertension Optimal Treatment (HOT) trial, diabetic patients whose diastolic blood pressure was equal or less than 80 mm Hg had a 51% reduction in major CV events compared with diabetic patients whose diastolic blood pressure was equal or less than 90 mm Hg [17].

**Figure 1:**
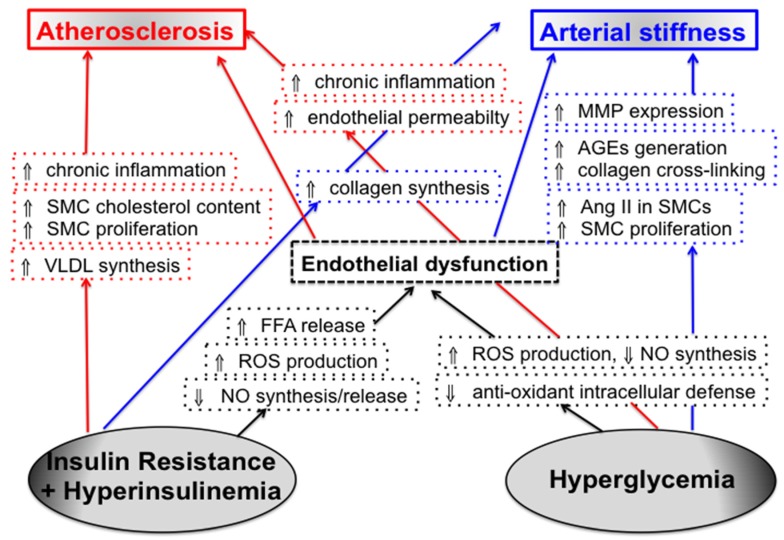
Pathophysiologic mechanisms through which insulin resistance and hyperglycemia may affect the arterial wall.

Although the role of chronic hyperglycemia in macrovascular disease and CV risk is not clearly established, there are no doubts that glucose may provoke structural and functional changes in the vascular wall by various mechanisms (Figure 1). Hyperglycemia has been shown to trigger endothelial dysfunction through decrease in nitric oxide (NO) synthesis, increase in free radicals levels, and deterioration of antioxidant defense mechanisms [18,19]. Chronic glycemic exposure also induces vascular SMCs proliferation and chronic inflammation [8,9], increases generation of advanced glycation end-products (AGEs) and enhances collagen cross-linking within the arterial wall [20], up-regulates matrix metalloproteinase-2 and -9 expression (enzymes degrading elastin) [21], augments the generation of angiotensin 2 in vascular tissue [22] and increases endothelial permeability [23]. Acute blood glucose fluctuations, reflecting the upward (post-prandial) and downward (inter-prandial) circadian shift of glucose levels, may further affect arterial wall homeostasis by triggering the oxidative stress and systemic inflammation and enhancing monocyte adhesion to the endothelium [24,25,26]. Described alterations may induce either arterial stiffening or early atherosclerotic changes or both. For example, endothelial dysfunction is considered a key event in the initiation of atherosclerotic process [27], yet it also leads to “functional” stiffening of arteries, as a continuous NO release by endothelium contributes to the functional regulation of arterial elasticity [28], aimed to adapt peripheral conduit artery mechanics to changes in blood flow.

## 3. Insulin Resistance

Insulin resistance, a primary biochemical abnormality in T2DM, is associated with a metabolic and CV cluster of disorders (central obesity, high blood pressure, dyslipidemia, hyperinsulinemia), each of which is an independent risk factor for CVD. Several prospective studies have demonstrated that insulin resistance, as assessed by various techniques, is related to CVD in both non-diabetic and diabetic subjects, independently of established risk factors. In the population of San Antonio Heart Study followed-up for 8 years, the risk of CVD events increased across quintiles of the homeostasis model assessment of insulin resistance (HOMA-IR) [29]; the association between HOMA-IR index and CVD was demonstrated also in a general population of the Bruneck Study, followed-up for 15 years [30]. In the elderly population of Uppsala, insulin resistance, as measured by the gold-standard method of euglycaemic insulin clamp, predicted coronary heart disease over a 10-year period [31].

Insulin receptors are present in endothelial cells, vascular SMCs and macrophages, yet the question whether the vascular insulin receptors contribute directly to the vascular pathology of metabolic insulin resistance is still open [32,33]. Insulin resistance has been shown to be associated with decreased synthesis/release of NO and enhanced generation of reactive oxygen species [34,35,36,37], as well as with an excessive free fatty acids release from adipose tissue. Increased circulating levels of free fatty acids may impair endothelial function [35,36,37] and induce a low-grade inflammation (through activation of nuclear factor kB) [36,37] (Figure 1). Hyperinsulinemia augments hepatic very-low-density lipoproteins synthesis, increases cholesterol transport/synthesis in cultured arterial SMCs, stimulates the proliferation of arterial SMCs, augments collagen synthesis and turns on multiple genes involved in inflammation [8,9,38,39,40].

## 4. Vascular Biomarkers

Diabetes-related vascular impairment can be already detected in a preclinical phase through vascular biomarkers. A “biomarker” was defined by the National Institutes of Health as a “characteristic that is objectively measured and evaluated as an indicator of normal biological processes, pathogenic processes, or pharmacologic responses to a therapeutic intervention” [41]. Therefore, biomarkers can be used to monitor the burden of subclinical disease in order to apply preventive measures, and they also enable to assess the response of subclinical disease to preventive/therapeutic interventions. Vascular biomarkers may be particularly informative, as they are capable to detect subclinical structural or functional impairment in different vascular beds [42]. Endothelial function, carotid intima-media thickness (CIMT) and arterial stiffness are the biomarkers more frequently used in the assessment of CV risk.

## 5. Endothelial Function

The endothelium regulates vascular homeostasis through number of vasoactive molecules, and a loss of normal endothelial function is believed a key event in the initiation of the atherosclerotic process [43]. Endothelial function can be measured by different techniques, yet the most widely applied technique is brachial artery flow-mediated dilation that allows appraising the endothelial function in a noninvasive way without the use of pharmacologic stimuli [44]. Due to its technical complexity and methodological shortcomings [42], this method is predominantly a research tool used to study the role of different risk factors in atherosclerotic process and to monitor the effect of therapeutic interventions.

Number of clinical studies have demonstrated an impaired endothelium-dependent vasodilation in conduit or resistance vessels of T2DM patients [45,46,47]; this impairment was related to plasma glucose, glucose levels fluctuation, HbA1c and insulin resistance [48,49,50], low-density lipoprotein size [46], serum concentration of AGEs [51], endothelial oxidative stress [52] and chronic inflammation [53]. Endothelial dysfunction seems to precede the development of diabetes, as impaired endothelium-dependent vasodilation was observed in healthy non-diabetic subjects who have a first degree relative with T2DM [47], as well as in subjects with impaired glucose tolerance [47,48].

## 6. CIMT and Plaque Presence

CIMT is a combined measure of tunica intima and tunica media and is measured by high-resolution ultrasound in different segments of extracranial carotid tree as the distance between the intima-luminal and the medial-adventitial interfaces. CIMT and carotid plaques are considered surrogate measures of atherosclerosis, and have been shown to be associated with CV risk factors and CV outcomes [54,55]. Increased CIMT reflects very early atherosclerotic changes, whereas plaque presence indicates more advanced atherosclerotic process. Both CIMT and plaques can be measured during a single ultrasound examination and provide complementary prognostic information. Indeed, the American Society of Echocardiography consensus statement specified that carotid-artery ultrasonography for CV disease risk prediction should be based on a thorough scan of the extracranial carotid tree to detect the presence of plaques, followed by the measurement of CIMT in the common carotid artery (CCA) [56]. New advances in ultrasound, like accurate semi-automatic radiofrequency-based CIMT measurement [57] and 3-D-based plaque volume estimation [58], might further improve the accuracy, reproducibility and interpretation of carotid measures, and thus refine their predictive value [42].

T2DM patients have higher CIMT (on average by 130 μm), higher prevalence of carotid plaques and higher plaque volume as compared to controls [59,60,61], and CIMT and carotid plaque prevalence have been shown to be associated with fasting plasma glucose levels, glucose fluctuation or HbA1c, both in non-diabetic and in diabetic populations [62,63,64,65,66]. Within T2DM patients, a 1 SD difference in fasting glucose (3.2 mmol/L) was associated with a 26-μm thicker CIMT [64], and each 1% increase in HbA1c or each year of T2DM duration were associated with a 35% or 33% increased odds of a thicker CIMT in CCA or in bulb, respectively [65]. However, increase in plasma glucose levels is only a part of more complex metabolic impairment, and the association between CIMT and glucose exposure should be adjusted for other components of metabolic syndrome, like obesity, hypertension and dyslipidemia. Indeed, recent data from a large population-based study have shown that neither fasting glucose, nor 2-h glucose, nor HbA1c were associated with CIMT when adjusted to sex, age, hypertension and waist circumference [67]. This observation supports the premise that plasma glucose is a risk factor for atherosclerosis, but probably of minor importance than traditional risk factors or other components of metabolic syndrome [8,9].

Studies evaluating the impact of insulin resistance and fasting plasma insulin levels on carotid wall thickness are not conclusive, probably due to differences in the methods used for insulin resistance estimation, differences in population studied and different adjustment for possible confounders. In the Atherosclerosis Risk in Communities (ARIC) study, fasting insulin levels were associated to mean CIMT, however, the model was not adjusted for abdominal obesity and triglycerides [68]. In the Insulin Resistance Atherosclerosis Study (IRAS), insulin sensitivity was negatively associated with CIMT, and this effect was partly explained by traditional CV risk factors, glucose tolerance and adiposity [69]. In the Malmo study, the association between HOMA-IR index and CIMT in non-diabetic subjects was fully explained by established cardiovascular risk factors, above all by hypertension [70], and in the Salzburg Atherosclerosis Prevention program in subjects at High Individual Risk (SAPHIR) study [71], the relationship between HOMA-IR index and carotid atherosclerosis was mostly dependent on the clustered expression of the components of the metabolic syndrome. Finally, in a healthy European population of the Relationship between Insulin Sensitivity and Cardiovascular Risk (RISC) study, the association between lower insulin sensitivity as measured by euglycemic hyperinsulinemic clamp and CIMT was observed only in men, and was mediated by circulating free fatty acids and adipocytokines [72]. In the women of the RISC study, CIMT was independently associated with fasting plasma glucose levels. These results imply that insulin resistance *per se* has no strong influence on carotid atherosclerosis, and that its effect on carotid wall is mediated by other metabolic, cellular and hemodynamic abnormalities related to the insulin resistance syndrome and diabetes, like dyslipidemia, free fatty acids, adipocytokines, chronic inflammation and hypertension [72,73,74,75].

It should be considered that increased CIMT in diabetic patients might also reflect adaptive arterial remodeling in response to altered mechanical stimuli [76,77]. T2DM patients have increased large artery stiffness, diameter and pulsatile load [77,78,79], and these changes may lead to increase in circumferential wall stress and pulsatile strain. Previous works have demonstrated a mutual adjustment between carotid wall thickness and luminal diameter aimed to maintain wall stress within homeostatic targets [80,81], as well as an independent relationship between CIMT and local pulse pressure [82], confirming the contribution of chronic cyclic stretching to arterial remodeling [83].

## 7. Arterial Stiffness

The aorta and large arteries transform the pulsatile flow generated by ventricular contraction into a continuous flow at the periphery. This cushioning function depends on the mechanical properties of the arterial wall that are mainly determined by the composition and organization of ECM. Arterial stiffening is characterized by degenerative changes of ECM (elastin fatigue fracture, collagen deposition and cross-linking), but also by alterations of vascular endothelial cells and SMCs. Diabetes may induce arterial stiffening through number of mechanisms related both to hyperglycemia and insulin resistance (Figure 1).

Multiple approaches to arterial stiffness assessment are now available for clinical use. Regional arterial stiffness estimates the propagation speed of the arterial pulse wave (pulse wave velocity, PWV) and is measured directly, as a ratio of distance between two measurement points divided by the time required for the pressure wave to travel this path. Carotid-femoral PWV (Cf-PWV) reflects above all the aortic stiffness and represents a gold standard for arterial stiffness measurement [84]. Brachial-ankle PWV (Ba-PWV) measures a pulse wave propagation speed over a longer arterial length including also muscular segments; this method is being primarily used in Asian countries. Local arterial stiffness, as assessed by radiofrequency-based ultrasound, describes the changes in arterial diameter/volume during the cardiac cycle for the corresponding change in distending pressure (*i.e.*, pulse pressure) [85]. In large European populations, normal and reference values according to age and blood pressure were established for Cf-PWV [86], and according to age and sex for local carotid and femoral distensibility [87,88]. These reference values are essential for the correct interpretation of stiffness measure in the individual subjects, as arterial distensibility is strongly age- and pressure-dependent, and the age-related increase in stiffness may differ between men and women [85]. Diabetes is supposed to accelerate the natural aging process of the arterial tree, *i.e.*, to induce more pronounced and earlier stiffening than expected for a given age [89,90]. Furthermore, a greater age-related stiffening of the aorta was described in diabetic women as compared to diabetic men [91], and this finding is in agreement with the observation that diabetes negates the protective effect of female sex and confers a greater relative risk in women than in men [2,92].

A predictive value of arterial stiffness for CVD has been clearly demonstrated. In a large meta-analysis, CVD events increased by 30% per 1 SD increase in log Cf-PWV [93], and in a recent prospective study, local stiffness of carotid and femoral artery was independently associated with CV events and all-cause mortality [94]. A large body of evidence has also demonstrated the association between diabetes, arterial stiffness and CV risk [6]. In the Hoorn study, in the Asklepios study and in a number of smaller studies, T2DM patients had significantly higher Cf-PWV or local carotid PWV as compared to healthy controls [77,78,79,95,96]. An increase in the indices of local carotid stiffness paralleled the increase in Cf-PWV in the Hoorn study [79] but not in the Asklepios study [78], suggesting that the impact of diabetes may differ in different parts of arterial tree [97]. Some data indicate that increased large artery stiffness appears already in prediabetic conditions. In a treatment-naïve and mostly “healthy” population from the ADDITION-Leicester cohort, Cf-PWV was increased in individuals with impaired fasting glucose or impaired glucose tolerance as compared to those with normal glucose metabolism, and the increase was identical to that of individuals with newly diagnosed T2DM [98]. These data are in line with the observation that increased Cf-PWV is an independent predictor of CV and overall mortality both in patients with T2DM and in subjects with impaired glucose tolerance, and that in both groups the mortality risk doubled when compared to controls [99].

Numbers of studies have also described the association between glycemic control, insulin resistance and arterial stiffness. In the ARIC study, indices of carotid stiffness increased with fasting plasma glucose, insulin and HbA1c [100,101], and in the Cardiometabolic Risk in Chinese Study, Cf-PWV increased with HbA1c [102]. In a middle-aged population of the Malmo Diet and Cancer study, fasting glucose, HOMA-IR index and HbA1c, together with waist circumference, triglycerides and HDL cholesterol were all predictors of Cf-PWV after a follow-up of 17 years [103]. In T2DM patients, stiffness of carotid artery was independently related to insulin sensitivity, measured by euglycemic-hyperinsulinemic clamp, and to duration of diabetes [104], as well as to HbA1c [77]. Ba-PWV was independently and positively associated with fasting plasma glucose, with one-hour post-challenge glucose or with HOMA-IR index in general population, in the pre-diabetic subjects, and in non-diabetic hypertensive subjects [105,106,107,108,109]. Finally, a recent study in middle-aged subjects free of CVD demonstrated the relationships of Cf-PWV with HOMA-IR index (direct) and telomere length (inverse), and suggested that insulin resistance linked with chronic inflammation can enhance telomere shortening (a marker of cellular senescence), and thus induce accelerated vascular aging [110].

The association between arterial stiffness and CV events or all-cause mortality can be explained, at least partly, by the adverse hemodynamic effect of arterial stiffening. In physiologic conditions, there is a stiffness gradient from proximal, distensible elastic arteries to distal, muscular arteries, which contributes to the wave reflection phenomenon. In a stiff arterial tree, the speed of propagation of the arterial pulse through the aorta is increased, and the increased speed of the forward traveling wave implies an earlier reflection of backward traveling wave from the periphery. Thus, the backward waves arrives to ascending aorta in systole instead of in diastole, and this shift in timing leads to an augmentation of aortic systolic blood pressure and pulse pressure and to a decrease in diastolic coronary perfusion pressure [111]. As a result, left ventricular afterload increases, together with myocardial workload, myocardial mass and oxygen demand, and diastolic coronary perfusion pressure decreases together with myocardial oxygen delivery. Moreover, the stiffness gradient between proximal elastic arteries and more distal muscular arteries decreases, and therefore, increases the transmission of pressure to the microcirculation that may be already damaged by diabetic microvascular disease.

## 8. Conclusions

A great amount of data demonstrates that hyperglycemia and insulin resistance activate number of mechanisms triggering the structural and functional changes in the arterial wall, which are likely to contribute to accelerated vascular aging and increased CV risk in T2DM. Clinical data evaluating the association between impaired glucose metabolism and vascular biomarkers of atherosclerosis suggest that hyperglycemia and/or insulin resistance *per se* had only a minor impact on atherosclerotic process when compared to traditional risk factors. On the other hand, accelerated arterial stiffening seems a hallmark of impaired glucose metabolism, and aortic stiffness results an independent predictor for CV and overall mortality not only in patients with T2DM but already in subjects with impaired glucose tolerance. Moreover, T2DM is associated with other metabolic and systemic abnormalities, like atherogenic dyslipidemia, hypertension and obesity that may cause atherosclerosis, arterial stiffening or both. Vascular biomarker are valuable in the diabetes research; they may help in identifying the mechanisms through which T2DM affects the vascular wall and induces CV complications, and they can be used to test the effect of therapeutic/life-style interventions on preclinical atherosclerosis and arterial stiffening. The clinical value of vascular biomarkers in CV risk estimation of diabetic patients is still under discussion. It should be considered that not every diabetic patient has the same metabolic phenotype, *i.e.*, carries the same spectrum of metabolic abnormalities. Therefore, the assessment of vascular age by means of vascular biomarkers might provide an integrated insight on diabetes-related vascular impairment of each patient and facilitate a personalized approach to the prevention and treatment. Finally, recent evidence suggests that childhood and adolescence are particularly vulnerable periods of life to the effects of cardiometabolic risk and later development of atherosclerosis and diabetes [112]. Screening of children and adolescents at high cardiometabolic risk (obese, off-springs of diabetic parents) and assessing the impact of lifestyle interventions on vascular biomarkers might help to mitigate CV complications in adulthood [113,114].

## References

[1] Beckman J.A., Creager M.A., Libby P. (2002). Diabetes and atherosclerosis: Epidemiology, pathophysiology, and management. JAMA.

[2] Kannel W.B., McGee D.L. (1979). Diabetes and cardiovascular risk factors: The Framingham study. Circulation.

[3] Booth G.L., Kapral M.K., Fung K., Tu J.V. (2006). Relation between age and cardiovascular disease in men and women with diabetes compared with non-diabetic people: A population-based retrospective cohort study. Lancet.

[4] Whiteley L., Padmanabhan S., Hole D., Isles C. (2005). Should diabetes be considered a coronary heart disease risk equivalent? Results from 25 years of follow-up in the Renfrew and Paisley survey. Diabetes Care.

[5] Colwell J.A., Lopes-Virella M., Halushka P.V. (1981). Pathogenesis of atherosclerosis in diabetes mellitus. Diabetes Care.

[6] Stehouwer C.D., Henry R.M., Ferreira I. (2008). Arterial stiffness in diabetes and the metabolic syndrome: A pathway to cardiovascular disease. Diabetologia.

[7] Stratton L.M., Adler A.J., Neil H.A., Matthews D.R., Manley S.E., Cull C.A., Hadden D., Turner R.C., Holman R.R. (2000). Association of glycaemia with macrovascular and microvascular complications of type 2 diabetes (UKPDS 35): Prospective observational study. BMJ.

[8] Bonfeld K.E., Tabas I. (2011). Insulin resistance, hyperglycemia and atherosclerosis. Cell Metabolism.

[9] DeFronzo R.A. (2010). Insulin resistance, lipotoxicity, type 2 diabetes and atherosclerosis: The missing links. The Claude Bernard Lecture 2009. Diabetologia.

[10] Zhang Y., Hu G., Yuan Z., Chen L. (2012). Glycosylated hemoglobin in relationship to cardiovascular outcomes and death in patients with type 2 diabetes: A systematic review and meta-analysis. PLoS ONE.

[11] Cavender M.A., Scirica B.M., Raz I., Steg P.G., McGuire D.K., Leiter L.A., Hirshberg B., Davidson J., Cahn A., Mosenzon O. (2015). Cardiovascular outcomes of patients in SAVOR-TIMI 53 by baseline hemoglobin A1c. Am. J. Med..

[12] Holman R.R., Paul S.J., Bethel M.A., Matthews D.R., Neil A.W. (2008). 10-Year follow-up of intensive glucose control in type 2 diabetes. N. Engl. J. Med..

[13] Dluhy R.G., McHanon G.T. (2008). Intensive glycemic control in the ACCORD and ADVANCE trials. N. Engl. J. Med..

[14] Gaede P., Vedel P., Larsen N., Jensen G.V., Parving H.H., Pedersen O. (2003). Multifactorial intervention and cardiovascular disease in patients with type 2 diabetes. N. Engl. J. Med..

[15] Costa J., Borges M., David C., Vaz Carneiro A. (2006). Efficacy of lipid lowering drug treatment for diabetic and non-diabetic patients: Meta-analysis of randomised controlled trials. BMJ.

[16] UK Prospective Diabetes Study Group (1998). Tight blood pressure control and risk of macrovascular and microvascular complications in type 2 diabetes: UKPDS 38. BMJ.

[17] Hansson L., Zanchetti A., Carruthers S.G., Dahlöf B., Elmfeldt D., Julius S., Ménard J., Rahn K.H., Wedel H., Westerling S., HOT Study Group (1998). Effects of intensive blood-pressure lowering and low-dose aspirin in patients with hypertension: Principal results of the Hypertension Optimal Treatment (HOT) randomised trial. Lancet.

[18] Tessari P., Cecchet D., Vedovato M. (2010). Nitric oxide synthesis is reduced in subjects with type 2 diabetes and nephropathy. Diabetes.

[19] Maritim A.C., Sanders R.A., Watkins J.B. (2003). Diabetes, oxidative stress, and antioxidants: A review. J. Biochem. Mol. Toxicol..

[20] Sell D.R., Monnier V.M. (2012). Molecular basis of arterial stiffening: Role of glycation—A mini-review. Gerontology.

[21] Death A.K., Fisher E.J., McGrath K.C., Yue D.K. (2003). High glucose alters matrix metalloproteinase expression in two key vascular cells: Potential impact on atherosclerosis in diabetes. Atherosclerosis.

[22] Lavrentyev E.N., Estes A.M., Malik K.U. (2007). Mechanism of high glucose induced angiotensin II production in rat vascular smooth muscle cells. Circ. Res..

[23] Zhao X.Y., Wang X.F., Li L., Zhang L., Shen D.L., Li D.H., Jin Q.S., Zhang J.Y. (2015). Effects of high glucose on human umbilical vein endothelial cell permeability and myosin light chain phosphorylation. Diabetol. Metab. Syndr..

[24] Rizzo M.R., Barbieri M., Marfella R., Paolisso G. (2012). Reduction of oxidative stress and inflammation by blunting daily acute glucose fluctuations in patients with type 2 diabetes: Role of dipeptidyl peptidase-IV inhibition. Diabetes Care.

[25] Monnier L., Mas E., Ginet C., Michel F., Villon L., Cristol J.P., Colette C. (2006). Activation of oxidative stress by acute glucose fluctuations compared with sustained chronic hyperglycemia in patients with type 2 diabetes. JAMA.

[26] Azuma K., Kawamori R., Toyofuku Y., Kitahara Y., Sato F., Shimizu T., Miura K., Mine T., Tanaka Y., Mitsumata M. (2006). Repetitive fluctuations in blood glucose enhance monocyte adhesion to the endothelium of rat thoracic aorta. Arterioscler. Thromb. Vasc. Biol..

[27] Davignon J., Ganz P. (2004). Atherosclerosis: Evolving vascular biology and clinical implication. Role of endothelial dysfunction in atherosclerosis. Circulation.

[28] Kinley S., Creager M.A., Fukumoto M., Hikita H., Fang J.C., Selwyn A.P., Ganz P. (2001). Endothelium-derived nitric oxide regulates arterial elasticity in human arteries *in vivo*. Hypertension.

[29] Hanley A.J., Williams K., Stern M.P., Haffner S.M. (2002). Homeostasis model assessment of insulin resistance in relation to the incidence of cardiovascular disease: The San Antonio heart study. Diabetes Care.

[30] Bonora E., Kiechl S., Willeit J., Oberhollenzer F., Egger G., Meigs J.B., Bonadonna R., Muggeo M. (2007). Homeostasis model assessment predicts incident symptomatic cardiovascular disease in Caucasian subjects from the general population. The Bruneck Study. Diabetes Care.

[31] Zethelius B., Lithell H., Hales C.N., Berne C. (2005). Insulin sensitivity, proinsulin and insulin as predictors of coronary heart disease. A population-based 10-year, follow-up study in 70-year-old men using the euglycemic glucose clamp. Diabetologia.

[32] Fulton D.J.R. (2009). Mechanisms of vascular insulin resistance. A substitute Akt?. Circ. Res..

[33] Mathen K.J., Steinberg H.O., Baron A.D. (2013). Insulin resistance in the vasculature. J. Clin. Invest..

[34] Steinberg H.O., Chaker H., Leaming R., Johnson A., Brechtel G., Baron A.D. (1996). Obesity/insulin resistance is associated with endothelial dysfunction. Implications for the syndrome of insulin resistance. J. Clin. Invest..

[35] Cersosimo E., DeFronzo R.A. (2006). Insulin resistance and endothelial dysfunction: The road map to cardiovascular diseases. Diabetes Metab. Res. Rev..

[36] Barazzoni R., Zanetti M., Gortan Cappellari G., Semolic A., Boschelle M., Codarin E., Pirulli A., Cattin L., Guarnieri G. (2012). Fatty acids acutely enhance insulin-induced oxidative stress and cause insulin resistance by increasing mitochondrial reactive oxygen species (ROS) generation and nuclear factor-kappaB inhibitor (IkappaB)-nuclear factor-kappaB (NFkappaB) activation in rat muscle, in the absence of mitochondrial dysfunction. Diabetologia.

[37] Nigro J., Osman N., Dart A.M., Little P.J. (2006). Insulin resistance and atherosclerosis. Endocrine Review.

[38] Koopmans S.J., Kushwaha R.S., DeFronzo R.A. (1999). Chronic physiologic hyperinsulinemia impairs suppression of plasma free fatty acids and increases de novo lipogenesis in conscious normal rats. Metabolism.

[39] Nakao J., Ito H., Kanayasu T., Murota S. (1985). Stimulatory effect of insulin on aortic smooth muscle cell migration induced by 12-L-hydroxy-5, 8, 10, 14-eicosatetraenoic acid and its modulation by elevated extracellular glucose levels. Diabetes.

[40] Pfeifle B., Ditschuneit H. (1981). Effect of insulin on the growth of cultured arterial smooth muscle cells. Diabetologia.

[41] Biomarkers Definition Working Group (2001). Biomarkers and surrogate endpoints: Preferred definitions and conceptual framework. Clin. Pharmacol. Ther..

[42] Vlachopoulos C., Xaplanteris P., Aboyans V., Brodmann M., Cífková R., Cosentino F., de Carlo M., Gallino A., Landmesser U., Laurent S. (2015). The role of vascular biomarkers for primary and secondary prevention. A position paper from the European Society of Cardiology Working Group on peripheral circulation: Endorsed by the Association for Research into Arterial Structure and Physiology (ARTERY) Society. Atherosclerosis.

[43] Deanfield J.E., Halcox J.P., Rabelink T.J. (2007). Endothelial function and dysfunction: Testing and clinical relevance. Circulation.

[44] Lekakis J., Abraham P., Balbarini A., Blann A., Boulanger C.M., Cockcroft J., Cosentino F., Deanfield J., Gallino A., Ikonomidis I. (2011). Methods for evaluating endothelial function: A position statement from the European society of cardiology working group on peripheral circulation. Eur. J. Cardiovasc. Prev. Rehabil..

[45] Mäkimattila S., Liu M.L., Vakkilainen J., Schlenzka A., Lahdenperä S., Syvänne M., Mäntysaari M., Summanen P., Bergholm R., Taskinen M.R. (1999). Impaired endothelium-dependent vasodilation in type 2 diabetes. Relation to LDL size, oxidized LDL, and antioxidants. Diabetes Care.

[46] Watts G.F., O’Brien W.J., Silvester W., Millar J.A. (1996). Impaired endothelium-dependent and independent dilatation of forearm resistance arteries in men with diet-treated non-insulin-dependent diabetes. Clin. Sci..

[47] Caballero A.E., Arora S., Saouaf R., Lim S.C., Smakowski P., Park J.Y., King G.L., LoGerfo F.W., Horton E.S., Veves A. (1999). Microvascular and macrovascular reactivity is reduced in subjects at risk for type 2 diabetes. Diabetes.

[48] Vehkavaara S., Seppala-Lindroos A., Westerbacka J., Groop P.H., Yki-Järvinen H. (1999). *In vivo* endothelial dysfunction characterizes patients with impaired fasting glucose. Diabetes Care.

[49] Balletshofer B.M., Rittig K., Enderle M.D., Volk A., Maerker E., Jacob S., Matthaei S., Rett K., Häring H.U. (2000). Endothelial dysfunction is detectable in young normotensive first-degree relatives of subjects with type 2 diabetes in association with insulin resistance. Circulation.

[50] Torimoto K., Okada Y., Mori H., Tanaka Y. (2013). Relationship between fluctuations in glucose levels measured by continuous glucose monitoring and vascular endothelial dysfunction in type 2 diabetes mellitus. Cardiovasc. Diabetol..

[51] Tan K.C., Chow W.S., Ai V.H. (2002). Advanced glycation end products and endothelial dysfunction in type 2 diabetes. Diabetes Care.

[52] Su Y., Liu X.M., Sun Y.M., Jin H.B., Fu R., Wang Y.Y., Wu Y., Luan Y. (2008). The relationship between endothelial dysfunction and oxidative stress in diabetes and prediabetes. Int. J. Clin. Pract..

[53] Huang A.L., Vita J.A. (2006). Effects of systemic inflammation on endothelium-dependent vasodilation. Trend. Cardiovasc. Med..

[54] Polak J.F., Person S.D., Wei G.S., Godreau A., Jacobs D.R., Harrington A., Sidney S., O’Leary D.H. (2010). Segment-specific associations of carotid intima-media thickness with cardiovascular risk factors: The Coronary Artery Risk Development in Young Adults (CARDIA) study. Stroke.

[55] Touboul P.J., Hennerici M.G., Meairs S., Adams H., Amarenco P., Bornstein N., Csiba L., Desvarieux M., Ebrahim S., Hernandez Hernandez R. (2012). Mannheim carotid intima-media thickness and plaque consensus (2004-2006-2011). An update on behalf of the advisory board of the 3rd, 4th and 5th watching the risk symposia, at the 13th, 15th and 20th European Stroke Conferences, Mannheim, Germany, 2004, Brussels, Belgium, 2006, and Hamburg, Germany, 2011. Cerebrovasc. Dis..

[56] Stein J.H., Korcarz C.E., Hurst R.T., Lonn E., Kendall C.B., Mohler E.R., Najjar S.S., Rembold C.M., Post W.S., American Society of Echocardiography Carotid Intima-Media Thickness Task Force (2008). Use of carotid ultrasound to identify subclinical vascular disease and evaluate cardiovascular disease risk: A consensus statement from the American Society of Echocardiography Carotid Intima-Media Thickness Task Force. J. Am. Soc. Echocardiogr..

[57] Engelen L., Ferreira I., Stehouwer C.D., Boutouyrie P., Laurent S. (2013). Reference Values for Arterial Measurements Collaboration. Reference intervals for common carotid intima-media thickness measured with echotracking: Relation with risk factors. Eur. Heart J..

[58] Ainsworth C.D., Blake C.C., Tamayo A., Beletsky V., Fenster A., Spence J.D. (2005). 3D ultrasound measurement of change in carotid plaque volume: A tool for rapid evaluation of new therapies. Stroke.

[59] Brohall G., Odén A., Fagerberg B. (2006). Carotid artery intima-media thickness in patients with type 2 diabetes mellitus and impaired glucose tolerance: A systematic review. Diabet. Med..

[60] Mostaza J.M., Lahoz C., Salinero-Fort M.A., de Burgos-Lunar C., Laguna F., Estirado E., García-Iglesias F., González-Alegre T., Cornejo-Del-Río V., Sabín C. (2015). Carotid atherosclerosis severity in relation to glycemic status: A cross-sectional population study. Atherosclerosis.

[61] Pollex R.L., Spence J.D., House A.A., Fenster A., Hanley A.J.G., Zinman B., Harris S.B., Hegele R.A. (2005). A comparison of ultrasound measurements to assess carotid atherosclerosis development in subjects with and without type 2 diabetes. Cardiovasc. Ult..

[62] Verdoia M., Schaffer A., Cassetti E., Barbieri L., Di Ruocco M.V., Perrone-Filardi P., Marino P., de Luca G., On behalf of the Novara Atherosclerosis Study Group (2014). Glycosylated hemoglobin and coronary artery disease in patients without diabetes mellitus. Am. J. Prev. Med..

[63] Haring R., Baumeister S.E., Lieb W., von Sarnowski B., Volzke H., Felix S.B., Nauck M. (2014). Glycated hemoglobin as a marker of subclinical atherosclerosis and cardiac remodeling among non-diabetic adults from the general population. Diabetes Res. Clin. Pract..

[64] Wagenknecht L.E., D’Agostino R., Savage P.J., O’Leary D.H., Saad M.F., Haffner S.M. (1997). Duration of diabetes and carotid wall thickness. The Insulin Resistance Atherosclerosis Study (IRAS). Stroke.

[65] Shah A.S., Dolan L.M., Kimball T.R., Gao Z., Khoury P.R., Daniels S.R., Urbina E.M. (2009). Influence of duration of diabetes, glycemic control, and traditional cardiovascular risk factors on early atherosclerotic vascular changes in adolescents and young adults with type 2 diabetes mellitus. J. Clin. Endocrinol. Metab..

[66] Chen X.M., Zhang Y., Shen X.P., Huang Q., Ma H., Huang Y.L., Zhang W.Q., Wu H.J. (2010). Correlation between glucose fluctuations and carotid intima-media thickness in type 2 diabetes. Diabetes Res. Clin. Pract..

[67] Kowall B., Ebert N., Then C., Thiery J., Koenig W., Meisinger C., Rathmann W., Seissler J. (2012). Association between blood glucose and carotid intima-media thickness disappears after adjustment for shared risk factors: The KORA F4 study. PLoS ONE.

[68] Folsom A.R., Eckfeldt J.H., Weitzman S., Ma J., Chambless L.E., Barnes R.W., Cram K.B., Hutchinson R.G. (1994). Relation of carotid artery wall thickness to diabetes mellitus, fasting glucose and insulin, body size, and physical activity. Atherosclerosis Risk in Communities (ARIC) Study Investigators. Stroke.

[69] Howard G., O’Leary D.H., Zaccaro D., Haffner S., Rewers M., Hamman R., Selby J.V., Saad M.F., Savage P., Bergman R. (1996). Insulin sensitivity and atherosclerosis. The Insulin Resistance Atherosclerosis Study (IRAS) Investigators. Circulation.

[70] Hedblad B., Nilsson P., Janzon L., Berglund G. (2000). Relation between insulin resistance and carotid intima-media thickness and stenosis in non-diabetic subjects. Results from a cross-sectional study in Malmö, Sweden. Diabet. Med..

[71] Sourij H., Schmoelzer I., Dittrich P., Paulweber G., Iglseder B., Wascher T.C. (2008). Insulin resistance as a risk factor for carotid atherosclerosis. A comparison of the homeostasis model assessment and short insulin tolerance test. Stroke.

[72] Kozakova M., Natali A., Dekker J., Beck-Nielsen H., Laakso M., Nilsson P., Balkau B., Ferrannini E., RISC Investigators (2013). Insulin sensitivity and carotid intima-media thickness: Relationship between Insulin Sensitivity and Cardiovascular risk study. Arterioscler. Thromb. Vasc. Biol..

[73] Armstrong K.A., Hiremagular B., Haluska B.A., Campbell S.B., Hawley C.M., Marks L., Prins J., Johnson D.W., Isbel N.M. (2005). Free fatty acids are associated with obesity, insulin resistance, and atherosclerosis in renal transplant recipients. Transplantation.

[74] Hayaishi-Okano R., Yamasaki Y., Katakami N., Ohtoshi K., Gorogawa S., Kuroda A., Matsuhisa M., Kosugi K., Nishikawa N., Kajimoto Y. (2002). Elevated C-reactive protein associates with early-stage carotid atherosclerosis in young subjects with type 1 diabetes. Diabetes Care.

[75] Gardener H., Sjoberg C., Crisby M., Goldberg R., Mendez A., Wright C.B., Elkind M.S., Sacco R.L., Rundek T. (2012). Adiponectin and carotid intima-media thickness in the northern Manhattan study. Stroke.

[76] Henry R.M., Kostense P.J., Dekker J.M., Nijpels G., Heine R.J., Kamp O., Bouter L.M., Stehouwer C.D.A. (2004). Carotid arterial remodeling: A maladaptive phenomenon in type 2 diabetes but not in impaired glucose metabolism: The Hoorn study. Stroke.

[77] Kozakova M., Morizzo C., Bianchi C., Di Filippi M., Miccoli R., Paterni M., Di Bello V., Palombo C. (2014). Glucose-related arterial stiffness and carotid artery remodeling: A study in normal subjects and type 2 diabetes patients. J. Clin. Endocrinol. Metab..

[78] Chirinos J.A., Segers P., Gillebert T.C., de Buyzere M.L., van Daele C.M., Khan Z.A., Khawar U., de Bacquer D., Rietzschel E.R., On behalf of the Asklepios Investigators (2013). Central pulse pressure and its hemodynamic determinants in middle-aged adults with impaired fasting glucose and diabetes: The Asklepios study. Diabetes Care.

[79] Schram M.T., Henry R.M., van Dijk R.A., Kostense P.J., Dekker J.M., Nijpels G., Heine R.J., Bouter L.M., Westerhof N., Stehouwer C.D. (2004). Increased central artery stiffness in impaired glucose metabolism and type 2 diabetes: The Hoorn Study. Hypertension.

[80] Chironi G., Gariepy J., Denarie N., Balice M., Megnien J.L., Levenson J., Simon A. (2003). Influence of hypertension on early carotid artery remodeling. Atheroscler. Thromb. Vasc. Biol..

[81] Kozakova M., Palombo C., Paterni M., Anderwald C.H., Konrad T., Colgan M.P., Flyvbjerg A., Dekker J. (2008). Body composition and common carotid artery remodeling in a healthy population. J. Clin. Endocrinol. Metab..

[82] Winston G.J., Palmas W., Lima J., Polak J.F., Bertoni A.G., Burke G., Eng J., Gottesman R., Shea S. (2013). Pulse pressure and subclinical cardiovascular disease in the multi-ethnic study of atherosclerosis. Am. J. Hypertens..

[83] Boutouyrie P., Bussy C., Hayoz D., Hengstler J., Dartois N., Laloux B., Brunner H., Laurent S. (2000). Local pulse pressure and regression of arterial wall hypertrophy during long-term antihypertensive treatment. Circulation.

[84] Mancia G., Fagard R., Narkiewicz K., Redón J., Zanchetti A., Böhm M., Christiaens T., Cifkova R., de Backer G., Dominiczak A. (2013). 2013 ESH/ESC Guidelines for the management of arterial hypertension: the Task Force for the management of arterial hypertension of the European Society of Hypertension (ESH) and of the European Society of Cardiology (ESC). J. Hypertens..

[85] Kozakova M., Morizzo C., Guarino D., Federico G., Miccoli M., Giannattasio C., Palombo C. (2015). The impact of age and risk factors on carotid and carotid-femoral pulse wave velocity. J. Hypertens..

[86] The Reference Values for Arterial Stiffness Collaboration (2010). Determinants of pulse wave velocity in healthy people and in the presence of cardiovascular risk factors: “Establishing normal and reference values”. Eur. Heart. J..

[87] Engelen L., Bossuyt J., Ferreira I., van Bortel L.M., Reesink K., Segers P., Stehouwer C.D., Laurent S., Boutouyrie P., On behalf of the Reference Values for Arterial Measurements Collaboration (2015). Reference values for local arterial stiffness. Part A: Carotid artery. J. Hypertens..

[88] Bossuyt J., Engelen L., Ferreira I., Stehouwer C.D., Boutouyrie P., Laurent S., Segers P., Reesink K., van Bortel L.M., On behalf of the Reference Values for Arterial Measurements Collaboration (2015). Reference values for local arterial stiffness. Part B: Femoral artery. J. Hypertens..

[89] Nilsson P.M. (2014). Hemodynamic aging as the consequence of structural changes associated with Early Vascular Aging (EVA). Aging Dis..

[90] Najjar S.S., Scuteri A., Lakatta E.G. (2005). Arterial aging: Is it an immutable cardiovascular risk factor?. Hypertension.

[91] De Angelis L., Millasseau S.C., Smith A., Viberti G., Jones R.H., Ritter J.M., Chowienczyk P.J. (2004). Sex differences in age-related stiffening of the aorta in subjects with type 2 diabetes. Hypertension.

[92] Kannel W.B., Wilson P.W. (1995). Risk factors that attenuate the female coronary disease advantage. Arch. Intern. Med..

[93] Ben-Shlomo Y., Spears M., Boustred C., May M., Anderson S.G., Benjamin E.J., Boutouyrie P., Cameron J., Chen C.H. (2014). Aortic pulse wave velocity improves cardiovascular event prediction: An individual participant meta-analysis of prospective observational data from 17,635 subjects. J. Am. Coll. Cardiol..

[94] Van Sloten T.T., Schram M.T., van den Hurk K., Dekker J.M., Nijpels G., Henry R.M., Stehouwer C.D. (2014). Local stiffness of the carotid and femoral artery is associated with incident cardiovascular events and all-cause mortality: The Hoorn study. J. Am. Coll. Cardiol..

[95] Cameron J.D., Bulpitt C.J., Pinto E.S., Rajkumar C. (2003). The aging elastic arteries. A comparison of diabetic and nondiabetic subjects. Diabetes Care.

[96] Lacy P.S., Brien O., Stanley D.G., Deware A.G., Swales P.P.R., Williams B. (2004). Increased pulse wave velocity is not associated with elevated augmentation index in patients with diabetes. J. Hypertens..

[97] Paini A., Boutouyrie P., Calvet D., Tropeano A.I., Laloux B., Laurent S. (2006). Carotid and aortic stiffness. Determinants of discrepancies. Hypertension.

[98] Webb D.R., Khunti K., Silverman R., Gray L.J., Srinivasan B., Lacy P.S., Williams B., Davies M.J. (2010). Impact of metabolic indices on central artery stiffness: Independent association of insulin resistance and glucose with aortic pulse wave velocity. Diabetologia.

[99] Cruickshank K., Riste L., Anderson S.G., Wright J.S., Dunn G., Gosling R.G. (2002). Aortic pulse-wave velocity and its relationship to mortality in diabetes and glucose intolerance: An integrated index of vascular function?. Circulation.

[100] Rubin J., Nambi V., Chambless L.E., Steffes M.W., Juraschek S.P., Coresh J., Sharrett A.R., Selvin E. (2012). Hyperglycemia and arterial stiffness: The atherosclerosis risk in communities study. Atherosclerosis.

[101] Salomaa V., Riley W., Kark J.D., Nardo C., Folsom A.R. (1995). Non-insulin-dependent diabetes mellitus and fasting glucose and insulin concentration are associated with arterial stiffness indexes. The ARIC Study. Atherosclerosis Risk in Communities Study. Circulation.

[102] Liang J., Zhou N., Teng F., Zou C., Xue Y., Yang M., Song H., Qi L. (2012). Hemoglobin A1c levels and aortic arterial stiffness: The Cardiometabolic Risk in Chinese (CRC) study. PLoS ONE.

[103] Gottsäter M., Östling G., Persson M., Engström G., Melander O., Nilsson P.M. (2015). Non-hemodynamic predictors of arterials stiffness after 17 years follow-up: Malmö diet and cancer study. J. Hypertens..

[104] Emoto M., Nishizawa Y., Kawagishi T., Maekawa K., Hiura Y., Kanda H., Izumotani K., Shoji T., Ishimura E., Inaba M. (1998). Stiffness indexes beta of the common carotid and femoral arteries are associated with insulin resistance in NIDDM. Diabetes Care.

[105] Wang J., Liu L., Zhou Y., Wang C., Hu H., Hoff K., Guo Y., Gao X., Wang A., Wu S. (2014). Increased fasting glucose and the prevalence of arterial stiffness: A cross-sectional study in Chinese adults. Neurol. Res..

[106] Shin J.Y., Lee H.R., Lee D.C. (2011). Increased arterial stiffness in healthy subjects with high-normal glucose levels and in subjects with pre-diabetes. Cardiovasc. Diabetol..

[107] Niijima K., Muranaka Y., Ando T., Okada S., Niijima Y., Hashimoto K., Yamada M., Ohshima K., Mori M., Ono K. (2012). Elevated 1-h plasma glucose following 75-g oral glucose load is a predictor of arterial stiffness in subjects with normal glucose tolerance. Diabet. Med..

[108] Ho C.T., Lin C.C., Hsu H.S., Liu C.S., Davidson L.E., Li T.C., Li C.I., Lin W.Y. (2011). Arterial stiffness is strongly associated with insulin resistance in Chinese—A population-based study (Taichung Community Health Study, TCHS). J. Atheroscler. Thromb..

[109] Seo H.S., Kang T.S., Park S., Park H.Y., Ko Y.G., Choi D., Jang Y., Chung N. (2005). Insulin resistance is associated with arterial stiffness in nondiabetic hypertensives independent of metabolic status. Hypertens. Res..

[110] Strazhesko I., Tkacheva O., Boytsov S., Akasheva E., Vygodin V., Skvortsov D., Nilsson P. (2015). Association of insulin resistance, arterial stiffness and telomere length in adults free of cardiovascular diseases. PLoS ONE.

[111] Ikonomidis I., Makavos G., Lekakis J. (2015). Arterial stiffness and coronary artery disease. Curr. Opin. Cardiol..

[112] Agirbasli M., Tanrikulu A.M., Berenson G.S. (2015). Metabolic syndrome: Bridging the gap from childhood to adulthood. Cardiovasc. Ther..

[113] Kozakova M., Morizzo C., Bianchi V., Marchetti S., Federico G., Palombo C. (2016). Hedmodynamic overload and intra-abdominal adiposity in obese children: Relationship with cardiovascular structure and function. Nutr. Metabol. Cardiovasc. Dis..

[114] Jounala M., Raitakari M., Viikari J.S.A., Raitakari O.T. (2006). Obesity in youth is not an independent predictor of carotid IMT in adulthood. The Cardiovascular Risk in Young Finns Study. Atherosclerosis.

